# Exhaustion‐Resistant CD8 ^+^ T Cells in Ankylosing Spondylitis: A Proposed Three‐Axis Model

**DOI:** 10.1111/imm.70044

**Published:** 2025-10-02

**Authors:** Xuhong Zhang, Lu Jia, Xueni Lin, Lamei Zhou

**Affiliations:** ^1^ Department of Rheumatology Wuxi Affiliated Hospital of Nanjing University of Chinese Medicine Wuxi China

**Keywords:** CD8+ T cells, co‐stimulation, exhaustion resistance, gut–joint axis, HLA‐B27, IL‐15, T‐cell exhaustionankylosing spondylitis

## Abstract

Ankylosing spondylitis (AS) is a chronic immune‐mediated disease marked by sustained joint inflammation and aberrant bone remodelling. Although chronic antigen exposure usually enforces terminal exhaustion, emerging evidence indicates that a subset of CD8^+^ T cells in AS evades canonical exhaustion programmes while expressing programmed cell death protein 1 (PD‐1). These exhaustion‐resistant cells retain effector function and likely contribute to persistent tissue inflammation and structural damage. In this review, we dissect the cellular and molecular basis of exhaustion resistance in AS CD8^+^ T cells and focus on the convergence of intermittent T cell receptor (TCR) stimulation, metabolic adaptation that preserves mitochondrial fitness, and co‐stimulatory inputs from interleukin‐15 (IL‐15) and CD28. We propose an integrated three‐axis model governing CD8^+^ T cell fate and functional persistence in the AS context shaped by human leukocyte antigen‐B27 (HLA‐B27) and the gut–joint axis. Clarifying these mechanisms refines current views of T cell dysfunction in chronic inflammation and highlights therapeutic strategies aimed at reprogramming pathogenic immunity in AS.

## Graphical abstract

Exhaustion‐resistant CD8^+^ T cells may sustain ankylosing spondylitis. A three‐axis model—intermittent T‐cell receptor engagement, IL‐15/CD28 co‐stimulation and maintained metabolic fitness—explains how PD‐1^high CD8^+^ T cells retain effector function and accumulate from gut to entheses, promoting inflammation and aberrant bone remodelling. This concept yields measurable biomarkers (e.g., pSTAT5, TOX‐programme scores, metabolic flux) and therapeutic avenues, such as JAK–IL‐15 pathway modulation.

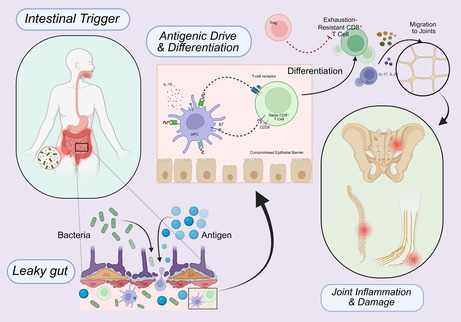

## 1 Introduction

Ankylosing spondylitis is an immune‐mediated inflammatory disease of the axial skeleton characterised by sacroiliac and spinal inflammation, fibrosis and osteoproliferation that can culminate in spinal fusion [1]. The disease typically begins in young adults and shows a strong genetic association with HLA‐B27 [2], which supports a central role for CD8^+^ T cells [3]. Beyond classical antigen presentation, HLA‐B27‐related phenomena, including misfolding‐induced unfolded protein response and interactions with killer cell immunoglobulin‐like receptors, link innate and adaptive compartments and help explain persistent inflammation [4, 5]. Biologics targeting tumour necrosis factor‐alpha (TNF‐α) or interleukin‐17 (IL‐17) improve symptoms but often fail to induce durable remission, indicating unresolved mechanisms [6, 7].

**FIGURE 1:**
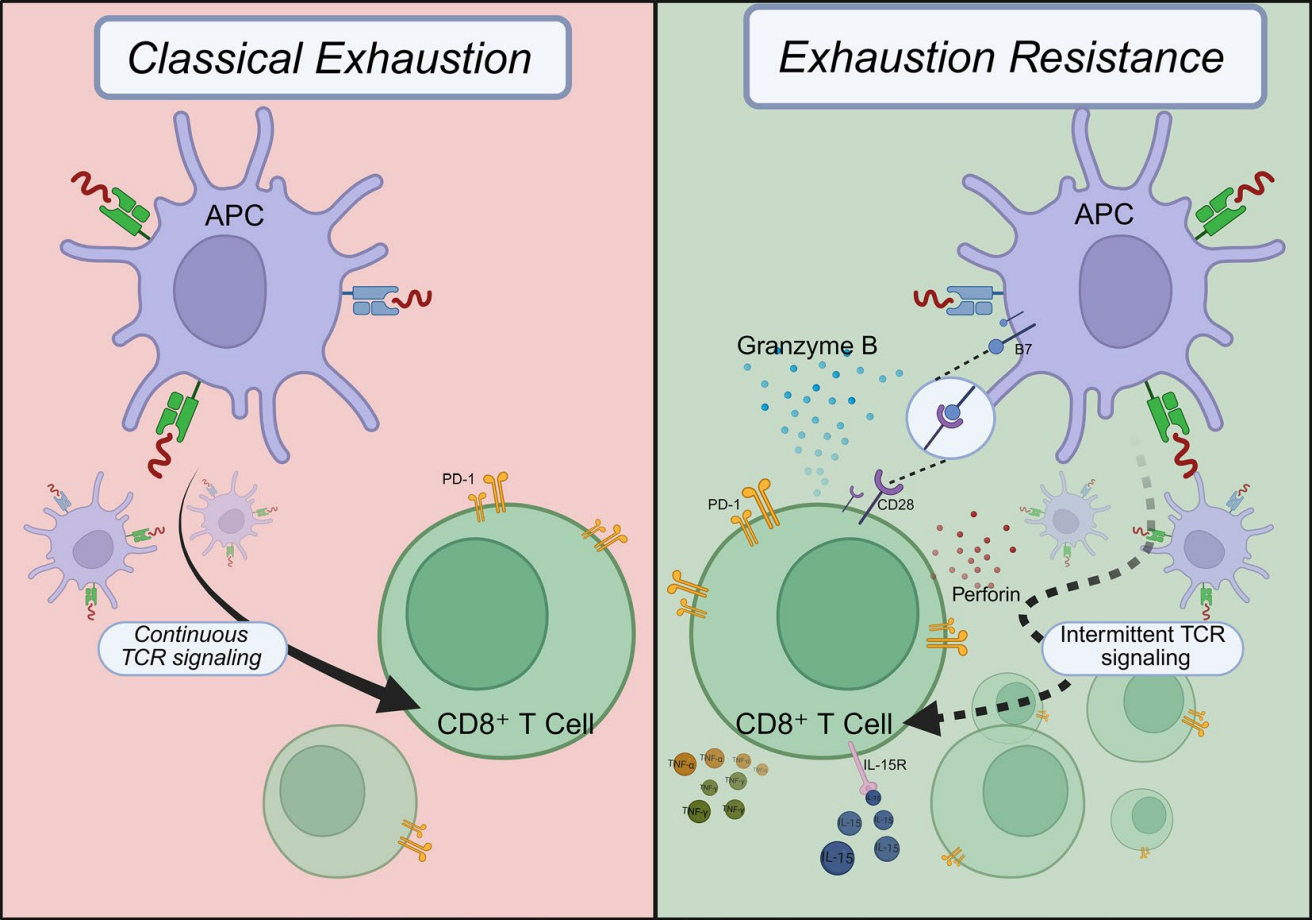
The paradox of CD8^+^ T‐cell exhaustion resistance in AS. Left: Classical CD8^+^ T‐cell exhaustion driven by continuous TCR signalling and characterised by progressive functional decline. Right: The exhaustion‐resistant phenotype observed in AS. In AS, intermittent antigenic stimulation together with CD28 co‐stimulation and interleukin‐15 allows CD8^+^ T cells to retain robust effector function and proliferative capacity despite the expression of inhibitory receptors such as PD‐1. (Created with BioRender.com).

A consistent observation in AS is the presence of PD‐1^high CD8^+^ T cells that remain metabolically competent and cytotoxic [8, 9]. In chronic antigenic stimulation, CD8^+^ T cells may enter a state termed T‐cell exhaustion, characterised by progressive loss of effector function together with defined transcriptional and epigenetic programmes [10]. Rather than representing canonical exhaustion, these cells appear to resist the TOX‐driven programme and undergo clonal expansion at inflamed sites [11, 12], which is consistent with intermittent stimulation by self or microbial antigens [13]. This central paradox is visually summarised in Figure 1.

Several AS‐specific features in AS help explain why this phenotype emerges and why our model is applicable. HLA‐B27 biology together with the ‘enthesis organ’ architecture creates niches for local immune activation; human enthesis contains resident lymphocytes that respond to interleukin‐23 and γδ T cells capable of interleukin‐17 production, supporting site‐restricted stimulation [14, 15, 16]. In parallel, a gut–joint axis is evident: subclinical intestinal inflammation is frequent in spondyloarthritis and can be tracked by faecal calprotectin, and an AS‐associated ileal microbial signature has been described [17, 18, 19]. Repetitive biomechanical stress at entheses provides episodic danger cues and antigen release, favouring intermittent and tissue‐confined TCR engagement rather than uniform, chronic exposure [20].

Against this backdrop, we advance a three‐axis framework that comprises intermittent TCR engagement, sustained metabolic fitness and IL‐15/CD28 co‐stimulation to account for exhaustion resistance and to generate testable predictions. We then integrate this framework with established AS pathways, including the interleukin‐23 (IL‐23)/IL‐17 circuit, ILC3 activity and gut barrier dysfunction, and we outline translational implications.

## 2 Classical Model of CD8 ^+^ T‐Cell Exhaustion

In acute infections, most effector CD8^+^ T cells undergo apoptosis after antigen clearance, with only a few differentiating into memory cells [21]. In chronic infections or cancer, however, continuous antigen exposure prevents CD8^+^ T cells from entering quiescence or forming long‐lived memory, instead driving them into a functionally constrained ‘exhausted’ state [22, 23]. Classically exhausted CD8^+^ T cells exhibit a characteristic phenotype and functional impairment: they co‐express multiple inhibitory receptors (e.g., PD‐1, CTLA‐4, LAG‐3, TIM‐3) [24], downregulate memory‐associated molecules, like IL‐7Rα (CD127) and CD62L, show reduced proliferative capacity, and undergo a hierarchical loss of effector functions (sequentially losing the ability to produce IL‐2, TNF‐α, then IFN‐γ) [25, 26]. At the transcriptional and epigenetic level, exhausted T cells establish a programme distinct from effector or memory T cells [27], governed by key regulators, such as T‐bet, Eomes, the NR4A family and TOX, among others, which collectively drive and maintain the exhausted cell fate. For example, the transcription factor TOX has been identified as essential for inducing and reinforcing the exhaustion programme—in its absence, CD8^+^ T cells in chronic infection fail to establish a stable exhausted phenotype [28]. These molecular mechanisms ensure the relative irreversibility of exhaustion, tightly regulating T‐cell responses under prolonged antigenic stimulation. Mechanistically, sustained TCR engagement in chronic settings activates the calcineurin–nuclear factor of activated T cells (NFAT) pathway in an altered mode (often unopposed by activator protein‐1 (AP‐1)), biasing gene expression towards an exhaustion programme. NFAT thereby induces transcription factors like TOX and NR4A that execute and lock in the exhaustion state [29, 30]. TOX in particular drives extensive epigenomic remodelling of T cells, cementing the exhausted phenotype. Consistently, without TOX, chronically stimulated CD8^+^ T cells show markedly reduced PD‐1 upregulation and retention of greater effector function despite persistent antigen exposure, underscoring TOX's central role in stabilising exhaustion.

From an evolutionary perspective, T‐cell exhaustion is viewed as a ‘necessary compromise’ – a trade‐off that sacrifices some pathogen‐clearing efficacy in chronic infection or cancer in exchange for protection of the host's own tissues [23]. By limiting T‐cell–mediated tissue damage, exhaustion is protective: for instance, in chronic viral infection models, the absence of negative regulators like PD‐1 leads to lethal immunopathology due to unchecked T cell activity [31]. Similarly, in autoimmunity, the exhaustion programme helps prevent self‐reactive T cells from inflicting rampant damage on host tissues [32]. The cost of this protection is attenuated immunity—exhausted CD8^+^ T cells are less able to eliminate persistent antigens (such as chronic HBV/HCV or tumour antigens), allowing pathogen persistence or tumour progression [27]. Recent studies further reveal that the exhausted T cell pool is heterogeneous, comprising subsets at different differentiation stages [33, 34]. Among these, progenitor or ‘stem‐like’ exhausted T cells (typically TCF‐1^+^ PD‐1^+^) retain some proliferative and differentiative capacity; they can self‐renew and give rise to partially re‐functionalised progeny, and they are the main subset reinvigorated by therapies like PD‐1 checkpoint blockade [35]. In contrast, terminally exhausted T cells (e.g., TIM‐3^+^ PD‐1^+^ TOX^high cells) are deeply dysfunctional, with minimal proliferative potential and resistance to reactivation [28]. In sum, the classical model describes a state of CD8^+^ T cells under chronic antigenic stress that is constrained by multiple inhibitory signals and locked in by transcriptional regulators. This state dampens T cell effector functions to strike a balance between pathogen clearance and prevention of host tissue damage.

## 3 Non‐Classically Exhausted CD8 ^+^ T Cells in AS

In this review, we use ‘exhaustion‐resistant’ to denote CD8^+^ T cells that (i) express one or more checkpoint receptors (most commonly PD‐1) and (ii) fail to engage the canonical TOX‐driven exhaustion programme (low or absent TOX with preserved TCF7/effector modules) and (iii) retain cytotoxic function and proliferative potential. Consistent with prior work, PD‐1 expression alone is not sufficient to infer exhaustion or exhaustion resistance.

Although T‐cell exhaustion is well‐established in infections and cancer, recent evidence indicates that atypical CD8^+^ T cell states exist in chronic inflammatory diseases, including autoimmune arthritis [36]. These T cells express certain ‘exhaustion’ markers on the surface but do not exhibit functional impairment; instead, they remain immunologically active. In juvenile idiopathic arthritis (JIA), for example, Petrelli et al. reported an accumulation of PD‐1^+^ CD8^+^ T cells in synovial fluid [36]; transcriptomic and functional assays showed that these cells were metabolically active cytotoxic effectors, but TOX was not assessed in that data set. We therefore do not classify these cells as exhaustion‐resistant by our definition; rather, they illustrate that checkpoint expression can occur outside terminal exhaustion in inflamed joints. Notably, similar PD‐1^+^ CD8^+^ subsets have been observed in lesional tissues of other chronic inflammatory diseases [34], again without uniform evidence for engagement of the TOX‐driven programme. In this review we reserve the term ‘exhaustion‐resistant’ for CD8^+^ T cells that co‐express checkpoint receptors yet show low or absent TOX together with retained effector function (see AS data sets cited herein) [24]. By contrast, in ankylosing spondylitis, we and others observe PD‐1^high CD8^+^ T cells with low or absent TOX and retained effector function, including clonal expansion at lesion sites, which satisfies our operational criteria for exhaustion resistance [8, 11, 12].

Studies in AS have reported analogous findings. In the peripheral blood and joint compartments of active AS patients, a distinct subset of CD8^+^ cytotoxic T lymphocytes (CTLs) has been identified that co‐expresses high levels of checkpoint receptors PD‐1, TIGIT and LAG‐3, yet still retains hallmark CTL functional capacities [8]. In a multi‐omics single‐cell analysis, Tang et al. found that PD‐1^+^ TIGIT^+^ CD8^+^ T cells in AS synovial fluid markedly downregulated the IL‐7 receptor α‐chain (CD127) but, upon stimulation, could still produce substantial IFN‐γ and TNF‐α and expressed cytolytic molecules like granzyme B and perforin. These cells also highly expressed tissue‐residency markers, such as CD103 and CD69, indicating their localisation at inflammatory sites [37]. Emerging evidence suggests a potential intrinsic link between TRM programmes and exhaustion‐resistance mechanisms, where TRM‐associated transcriptional networks (such as Hobit and Blimp‐1) may concurrently enhance tissue residency and limit exhaustion programming. Further elucidation of this link could substantially refine our understanding of CD8^+^ T‐cell persistence in chronic inflammation [38]. Crucially, single‐cell RNA sequencing revealed that this subset downregulated exhaustion‐associated genes and, most notably, the master transcription factor TOX, which is essential for establishing the canonically exhausted state in chronic infection or cancer [28]. The failure to upregulate TOX, possibly due to a distinct pattern of NFAT‐dominant signalling in the absence of AP‐1 in the AS microenvironment, likely prevents the stable epigenetic remodelling required for deep exhaustion, allowing these cells to remain in a highly functional state. Flow cytometry confirmed that these PD‐1^high CTLs were largely TOX^−^. Taken together, these findings support the existence of an exhaustion‐resistant CD8^+^ T‐cell subset in AS, in which inhibitory receptor expression coexists with preserved effector programmes (e.g., IFN‐γ, perforin). The apparent failure of the PD‐1 checkpoint to impose terminal exhaustion is more likely attributable to overriding pro‐inflammatory signals in the tissue microenvironment, as discussed below.

Notably, these exhaustion‐resistant CD8^+^ T cells tend to accumulate in lesional tissue (e.g., within the synovium of AS joints), in contrast to classically exhausted T cells that are often enriched in secondary lymphoid organs. The presence of such cells offers a potential explanation for the chronicity of AS: a subset of pathogenic CD8^+^ T cells may evade the canonical exhaustion programme and remain persistently aggressive against autoantigens, thereby driving prolonged inflammation and tissue damage. This concept shifts the paradigm beyond the previously dominant focus on the Th17/IL‐17 axis [39] and motivates renewed attention to the role of CD8^+^ T cells in AS immunopathology.

## 4 Potential Molecular Mechanisms: TCR Axis, Metabolic Axis and IL‐15/CD28 Axis

Why do certain CD8^+^ T cells in AS evade classical exhaustion? Current hypotheses centre on the combined influence of three factors: TCR signalling, cellular metabolic status and the co‐stimulatory/cytokine milieu. In this section, we discuss how each of these three pathways may modulate T‐cell exhaustion versus functional maintenance, and we propose how their interplay could operate in AS.

### 4.1 TCR Signalling Axis

The intensity and pattern of TCR signalling under chronic antigen exposure is a primary determinant of T‐cell fate. In general, continuous high‐intensity TCR stimulation drives the initiation of the exhaustion programme, but the intermittency and site‐specificity of stimulation can alter this outcome [28]. In AS, the autoantigens triggering inflammation may not be uniformly present continuously; rather, they are closely linked to biomechanical stress and the gut microbiome. Biomechanical stress at the entheses not only generates local damage‐associated molecular patterns (DAMPs) that stimulate resident T cells, but has been shown in HLA‐B27 transgenic rats to amplify inflammation via antigen release under mechanical loading, thereby reinforcing intermittent TCR activation [40]. Meanwhile, certain gut microbial antigens presented by HLA‐B27 may mimic self‐peptides, making gut inflammation an ‘engine’ that fuels systemic immune responses. For example, recent findings show that expanded CD8^+^ T cell clones in AS often use the TRBV9 gene and can recognise HLA‐B27—presented peptides from microbes and self, implicating antigenic drive along a gut–joint axis [41, 42]. However, unlike in chronic viral infections where antigen persists continuously, antigenic stimulation in AS may be episodic. It is hypothesised that flares triggered by gut microbiota changes or mechanical injury create cycles of recurrent stimulation and brief respite. According to a model primarily derived from chronic viral infection studies, such intermittent antigen exposure mitigates deep T‐cell exhaustion [26]. In this context, AS‐pathogenic CD8^+^ T cells might avoid the unrelenting signal overload that locks cells into an exhausted fate. This remains a key hypothesis for AS, as direct validation through methods like antigen‐specific TCR tracking in relevant models is still needed. Intermittent antigen exposure might allow epigenetic resetting or memory differentiation, thereby avoiding sustained NFAT‐driven TOX induction [43]. Concurrently, during each flare, abundant local antigen‐presenting cells likely provide strong co‐stimulatory signals, activating T cells in an effector mode rather than routing them into dysfunction.

### 4.2 Metabolic Reprogramming Axis

The metabolic state of a T cell is a critical intrinsic factor determining its function and fate. Exhausted T cells typically exhibit metabolic dysfunction: reduced mitochondrial mass and function, impaired oxidative phosphorylation, and an over‐reliance on glycolysis with diminished energetic efficiency [44]. In contrast, T‐cell subsets that are exhaustion‐resistant tend to maintain better mitochondrial function and metabolic flexibility. For example, progenitor ‘stem‐like’ exhausted T cells sustain higher mitochondrial respiration and fatty acid oxidation, which is thought to support their longevity and self‐renewal capacity [33, 45]. In inflammatory settings, exhaustion‐resistant CD8^+^ T cells appear to exhibit a highly active metabolic profile [46]. For example, PD‐1^+^ CD8^+^ T cells in JIA synovial fluid were found to have elevated glycolytic and mitochondrial activity to meet the demands of continuous proliferation and effector function [36]. However, it must be stressed that this is an extrapolation, as direct metabolic profiling of AS synovial T cells is currently a critical knowledge gap. We hypothesise that AS‐resident CD8^+^ T cells share this metabolic robustness, a point that urgently requires validation through techniques like Seahorse metabolic flux analysis and targeted metabolomics. On the other hand, the metabolic conditions of the tissue microenvironment (such as availability of oxygen, glucose and other nutrients) also influence T‐cell fate [47]. Although chronic inflammation in the synovium entails some degree of hypoxia and nutrient competition, it may not be as extremely suppressive as the tumour microenvironment [48]. Importantly, certain factors in chronic inflammation can activate metabolism‐favouring pathways; for instance, pro‐inflammatory cytokines can enhance T cell glucose uptake and glycolysis via mTOR signalling, thereby sustaining effector functions. Notably, PD‐1 ligation attenuates the phosphoinositide 3‐kinase–AKT (protein kinase B)–mechanistic target of rapamycin (PI3K–AKT–mTOR) signalling pathway in T cells, downregulating glucose transporter and glycolytic enzyme expression and shifting metabolism towards fatty acid oxidation [49, 50]. In exhaustion‐prone settings, this metabolic brake limits T‐cell effector capacity [51]. However, the unexhausted PD‐1^high CD8^+^ T cells in AS likely bypass this brake—Concurrent inflammatory signals, particularly from IL‐15 and CD28‐mediated co‐stimulation, appear to sustain Akt/mTOR signalling despite high PD‐1 expression, thus preserving glucose uptake capacity and mitochondrial functionality, although direct evidence from AS‐specific analyses is warranted [52]. This metabolic robustness of PD‐1^+^ CD8^+^ effectors in AS mirrors similar observations in chronic arthritis, suggesting a preserved effector phenotype despite inhibitory signalling. Of course, there is a flip side: excessive mTOR activation and glycolytic bias might accelerate exhaustion, as studies show that mitochondrial dysfunction coupled with HIF‐1α–driven glycolytic reprogramming can hasten the transition of precursor exhausted T cells into a terminally exhausted state [45, 53]. Therefore, in AS, exhaustion‐resistant CD8^+^ T cells maintain an optimal metabolic equilibrium characterised by mitochondrial flexibility and balanced glycolytic flux—possessing sufficient fuel and bioenergetic support to maintain cytotoxic activity, yet avoiding the mitochondrial collapse that triggers irreversible dysfunction. Cytokines like IL‐7 and IL‐15 may aid in this balance by promoting mitochondrial homeostasis and metabolic fitness in memory/resident T cells.

### 4.3 IL‐15/CD28 Co‐Stimulation Axis

Beyond the TCR and metabolic factors, the cytokine and co‐stimulatory milieu is pivotal in steering T cell functional states. In inflamed AS joints, innate immune and stromal cells produce abundant common γ‐chain cytokines (like IL‐7, IL‐15) and express co‐stimulatory ligands (like CD80/86), providing ‘signal 3’ cytokine support and co‐stimulation to T cells. IL‐15, in particular, is homologous to IL‐2 but more geared towards supporting memory and resident T‐cell survival. IL‐15 can promote CD8^+^ T‐cell survival and proliferation via signal transducer and activator of transcription 5 (STAT5) and mTOR pathways without inducing overt exhaustion or activation‐induced cell death [54]. In a chronic infection mouse model, exogenous IL‐15 selectively expanded PD‐1^+^ TCF‐1^+^ progenitor exhausted cells far more than terminally exhausted cells, thereby enhancing the self‐renewal and maintenance of the former [55]. A similar effect was observed in human tumour‐infiltrating lymphocytes: IL‐15 preferentially expanded a stem‐like subset of PD‐1^+^ CD8^+^ T cells [56]. These findings suggest that in AS inflamed joints, abundant IL‐15 signalling might prevent CD8^+^ T cells from prematurely exhausting, sustaining them in a more memory‐like, functional state. Meanwhile, ongoing CD28/B7 co‐stimulation is another crucial factor for preserving T cell function. PD‐1's inhibitory effect is largely executed by blunting CD28 signalling; therefore, if antigen‐presenting cells (APCs) in the inflammatory milieu continuously deliver CD28 co‐stimulation, the suppressive impact of PD‐1 signalling is partly offset, allowing T cells to retain a degree of effector activity [52]. Mechanistically, PD‐1 recruits SHP‐2 phosphatase to dephosphorylate CD28 and its downstream signalling components, dampening PI3K/Akt activation [49]. Continuous B7:CD28 engagement can thus counteract PD‐1–mediated signalling blockade, preserving Akt/mTOR‐driven functionality of the T cell [57]. Indeed, chronic infection models show that absence of CD28 co‐stimulation causes accelerated T‐cell dysfunction [10], whereas sustained low‐level CD28 signals favour long‐term maintenance of antiviral T cells. In AS patients, although a CD28^−^ exhausted phenotype subset exists in circulation, the activated CTLs at the joint often still express co‐stimulatory molecules and receive helper signals [58, 59]. Furthermore, the influx of myeloid cells and dendritic cells in AS synovium provides robust B7:CD28 co‐stimulation [60, 61]. Collectively, the IL‐15 and CD28 axes constitute a critical counter‐regulatory network: IL‐15 sustains T‐cell survival through STAT5‐Bcl2 signalling, while CD28 co‐stimulation preserves PI3K‐mTOR activity, synergistically promoting T‐cell persistence and actively antagonising inhibitory signals imposed by markers like PD‐1, thereby shaping the non‐exhausted, high‐functionality state of CD8^+^ T cells in AS [55, 62].

## 5 Integration of the Three Pathways: A Unified Model

**FIGURE 2:**
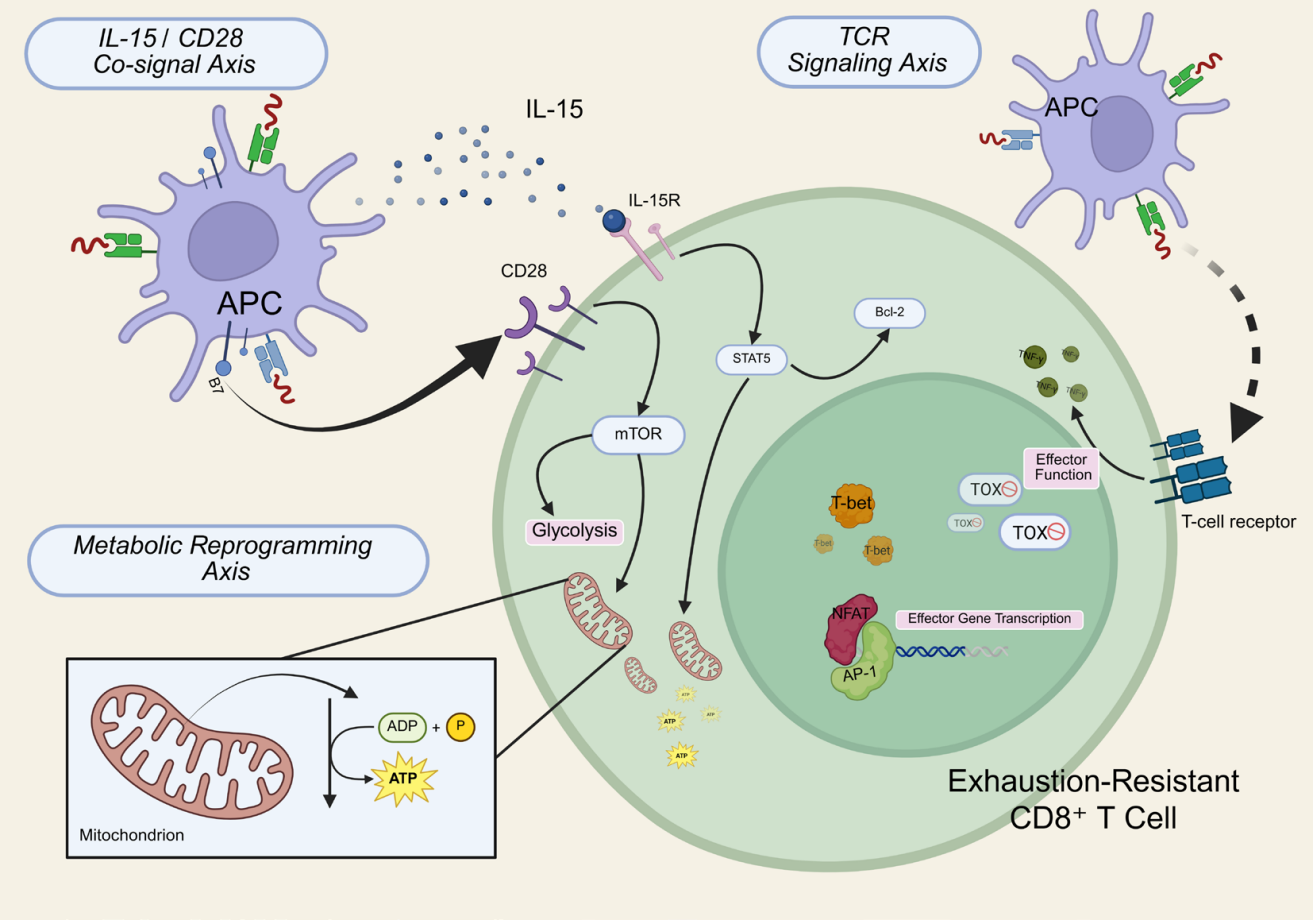
Integrated three‐axis model supporting exhaustion resistance. A mechanistic model illustrating how three interlinked axes synergise to preserve CD8^+^ T cell functionality in AS: (i) the IL‐15/CD28 co‐stimulation axis promotes survival and metabolic adaptation through STAT5 and mTOR signalling; (ii) the TCR signalling axis shows how intermittent stimulation supports effector gene transcription while preventing TOX‐mediated terminal exhaustion; (iii) the metabolic reprogramming axis highlights sustained mitochondrial fitness and glycolytic flexibility. (Created with BioRender.com).

We propose a mechanistically integrated model in which the exhaustion‐resistant phenotype of CD8^+^ T cells in AS emerges from the coordinated influence of three axes: persistent but intermittent TCR engagement, metabolic resilience and compensatory co‐stimulation through IL‐15 and CD28 (Figure 2). This triad forms a regulatory equilibrium between pro‐exhaustion cues and functional maintenance signals.

First, chronic but episodic antigenic stimulation—arising from biomechanical stress or gut‐derived antigens [63]—induces expression of inhibitory receptors like PD‐1, priming T cells towards an exhaustion trajectory [23]. However, in contrast to tumours or persistent viral infections, antigenic exposure in AS may be spatially and temporally confined, allowing partial reset of exhaustion programmes and epigenetic plasticity between flares.

Second, metabolic adaptability serves as a critical intracellular buffer. AS‐associated CD8^+^ T cells retain mitochondrial fitness and maintain moderate glycolytic flux, enabling sustained cytotoxic function while avoiding bioenergetic collapse [64, 65].

Third, the inflammatory synovial milieu in AS provides abundant CD28 co‐stimulation and common gamma‐chain (γc) cytokines such as IL‐15, which counterbalance PD‐1–mediated suppression by sustaining PI3K‐Akt–mTOR activity and upregulating pro‐survival programmes such as Bcl‐2 via STAT5. This extrinsic reinforcement promotes cellular persistence without functional attrition.

When these anti‐exhaustion signals collectively prevail, CD8^+^ T cells assume a ‘para‐exhausted’ or ‘functionally unexhausted’ state—characterised by surface expression of inhibitory receptors but lacking TOX‐driven epigenetic reprogramming. These cells maintain robust cytotoxicity, effector function and clonal persistence, thereby serving as long‐term contributors to inflammation and tissue injury in AS.

This model generates testable hypotheses: modulating the strength or synchronisation of these axes—e.g., reducing IL‐15 availability, inhibiting CD28 signalling or disrupting mitochondrial integrity—may shift CD8^+^ T cells towards terminal exhaustion and reduce pathogenicity. Such strategies may support future therapeutic designs that reprogram exhaustion resistance in AS‐specific immune circuits.

## 6 Integration With Other Immune Pathways in AS

**FIGURE 3:**
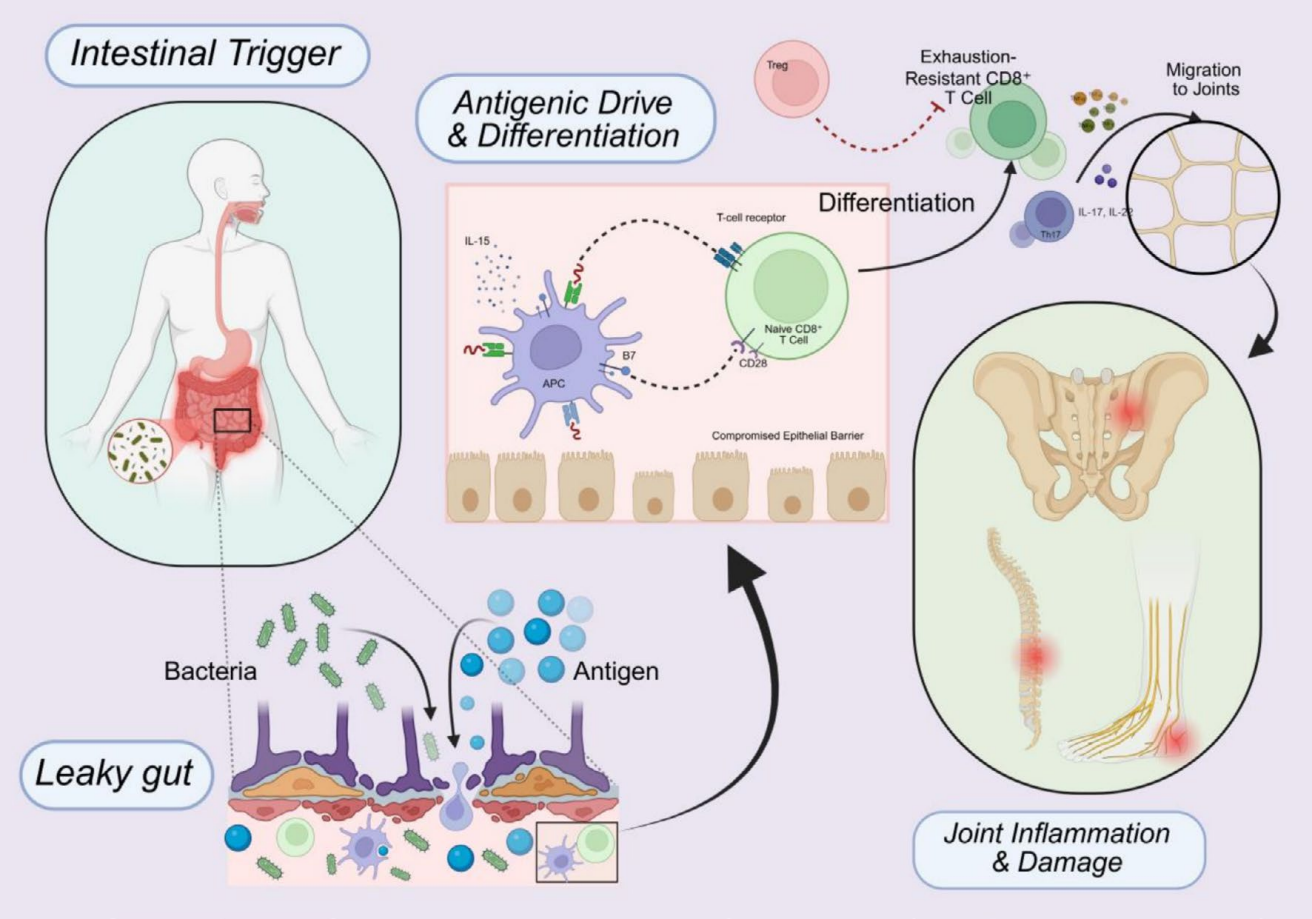
Role of exhaustion‐resistant CD8^+^ T cells in the gut–joint axis of AS. A schematic overview of AS pathogenesis centered on exhaustion‐resistant CD8^+^ T cells. Intestinal trigger: Disruption of the gut barrier permits microbial translocation. Antigenic drive and differentiation: Microbial and self‐antigens activate naive CD8^+^ T cells through antigen‐presenting cells (APCs) with interleukin‐15 (IL‐15)/CD28 co‐stimulation, promoting differentiation towards an exhaustion‐resistant phenotype. Migration to joints: These cells circulate and accumulate at inflamed entheses. Joint osteoimmune microenvironment: Exhaustion‐resistant CD8^+^ T cells contribute to inflammation and bone remodelling together with T helper 17 (Th17) cells, type 3 innate lymphoid cells (ILC3) and regulatory T cells (Tregs). (Created with BioRender.com.)

Placing our model of exhaustion‐resistant CD8^+^ T cells into the broader context of AS pathogenesis, we must consider its interplay with other well‐known immune pathways in the disease. AS is understood as a coordinated outcome of multiple cellular and molecular pathways, including an imbalance between T helper 17 (Th17) cells and regulatory T cells (Tregs) [66], an IL‐23/IL‐17 axis in which type 3 innate lymphoid cells (ILC3) are key contributors [67], and dysregulation of the gut microbiome together with impairment of the intestinal epithelial and vascular barriers [68, 69]. The presence of highly functional, exhaustion‐resistant CD8^+^ T cells adds a new dimension to this network and intersects with other pathways in several ways [8, 9]. This complex interplay, from the intestinal trigger to the multi‐cellular interactions within the joint microenvironment leading to pathology, is summarised in Figure 3.

### 6.1 Th17/IL‐23/IL‐17 Axis

A central pathogenic pathway in AS is the IL‐23–driven axis of Th17 cells and ILC3 producing IL‐17. IL‐17 and related cytokines (such as IL‐22) are crucial in joint inflammation and bone remodelling: IL‐17 promotes local inflammation and contributes to bone erosion [70], whereas IL‐22 is implicated in osteoproliferation [71]. These cytokines may disrupt the balance between bone resorption and formation, thereby driving both osteolysis and syndesmophyte formation—hallmarks of the paradoxical osteoimmune phenotype in AS. By secreting IFN‐γ, TNF‐α and IL‐22, exhaustion‐resistant CD8^+^ T cells in AS contribute to both osteoclastogenesis and enthesis ossification through the RANKL and BMP/Smad pathways. Separately, IL‐17–producing CD8^+^ T cells (Tc17) have also been described in AS. Whether these exhaustion‐resistant CD8^+^ T cells phenotypically or functionally overlap with the Tc17 population remains unresolved and represents a key direction for future investigation [72]. Future studies employing single‐cell multi‐omics analyses to co‐profile exhaustion markers (e.g., TOX), cytokine production (e.g., IL‐17, IFN‐γ) and TCR clonality in AS synovial fluid could elucidate this potential overlap. Meanwhile, IFN‐γ activates macrophages and synoviocytes to release additional chemokines and MMPs, amplifying inflammation and facilitating Th17 cell recruitment; TNF‐α synergises with IL‐17, jointly mediating inflammation and tissue destruction, which is why combined blockade of these cytokines yields enhanced clinical efficacy [73]. Moreover, IL‐17 can reciprocally affect CD8^+^ T cell activity; for example, IL‐17–driven inflammation can mature APCs and upregulate co‐stimulatory molecules, thereby boosting CD8^+^ T‐cell activation [74]. In sum, CD8^+^ T‐cell exhaustion‐resistance and the Th17/IL‐17 axis are not isolated—they mutually reinforce each other, acting as dual engines of chronic inflammation.

### 6.2 Treg Dysfunction

Regulatory T cells (Tregs) are crucial for maintaining immune tolerance, but their suppressive function appears compromised in AS [75]. While some studies report increased Treg proportions, these cells fail to suppress pathogenic T cells effectively [76]. Exhaustion‐resistant CD8^+^ T cells may explain this paradox, as they likely exhibit reduced sensitivity to Treg‐mediated suppression [77]. This resistance could be multifactorial. First, the strong pro‐survival and pro‐activation signals these cells receive, such as from IL‐15 via the STAT5 pathway, may make them inherently more resistant to suppressive signals. Second, the high levels of IFN‐γ they secrete can directly impair Treg function or render effector T cells less susceptible to Treg inhibition. Finally, while Tregs can use the PD‐1/PD‐L1 axis for suppression [78], this pathway is functionally antagonised in exhaustion‐resistant cells, blunting a key mechanism of Treg control. This creates a self‐reinforcing pathogenic circuit where dysfunctional Tregs fail to restrain hyperactive CD8^+^ T cells, whose inflammatory products in turn further limit Treg efficacy.

### 6.3 ILC3 and the Gut Axis

Innate lymphoid cells, especially ILC3, play a key role in the gut–joint axis of AS. ILC3 produce IL‐17 and IL‐22 in the gut mucosa and joints in response to IL‐23 [79]. HLA‐B27 transgenic rat studies show that a germ‐free environment prevents SpA‐like disease, whereas reintroduction of gut microbes triggers intestinal inflammation followed by arthritis [80]. This underscores the initiating role of the gut microbiome and barrier in AS. The CD8^+^ T‐cell exhaustion‐resistance concept complements this axis: the gut microbiota provides chronic but submaximal antigenic stimulation, activating certain clones of CD8^+^ T cells which then migrate to the joints. There, these T cells cooperate with cells like ILC3 to shape the inflammatory milieu. IL‐17/IL‐22 from ILC3 act on stromal cells to induce inflammatory mediators and drive bone changes, while IFN‐γ and TNF‐α from CD8^+^ T cells amplify this cytokine network. Conversely, cytokines from CD8^+^ T cells can affect barrier function: for example, IFN‐γ can increase gut epithelial permeability or alter microbiota composition, creating a ‘leaky gut’ and translocation of microbes that provide further T cell stimuli. This could explain why a significant fraction of AS patients have subclinical gut inflammation even without overt IBD.^19^ In essence, the persistence and hyperactivity conferred by the exhaustion‐resistant phenotype allow these CD8^+^ T cells to effectively link the ILC3–gut axis with the adaptive immune axis, forming a pivotal node in the pathogenic network of AS.

## 7 Testing the Proposed Model: An Experimental Roadmap

### 7.1 Samples and Comparators

Prioritise lesion‐site material whenever feasible, including synovial fluid, enthesis‐adjacent tissue and sacroiliac joint biopsies, with matched peripheral blood as a comparator. Pair AS cases with disease controls and healthy donors to define effect sizes.

### 7.2 Single‐Cell Multi‐Omics to Define Exhaustion Resistance

Use single‐cell RNA sequencing combined with surface‐protein indexing by cellular indexing of transcriptomes and epitopes by sequencing (CITE‐seq) to co‐measure transcriptomes and checkpoint or co‐stimulatory proteins in the same cells [81]. This enables an operational definition of exhaustion resistance as PD‐1^high cytotoxic CD8^+^ T cells with preserved effector programmes and low TOX‐module scores. Multiome profiling that measures gene expression and chromatin accessibility in the same cell can link resilience to accessible regulatory elements at loci such as TOX, TCF7 and STAT5 targets [82, 83]. Recommended outputs include gene‐module scores, chromatin peak enrichment, and joint clustering of RNA and ATAC features [84].

### 7.3 Spatial Context of Sustaining Signals

Apply spatial transcriptomics on sacroiliac or enthesis sections to localise IL‐15, B7 ligands and antigen‐presenting niches relative to PD‐1^high CD8^+^ T cell aggregates [85]. Report spatial co‐enrichment statistics and neighbourhood analyses to identify microenvironments that could maintain effector competence [86].

### 7.4 TCR Tracking and Evidence for Intermittent Stimulation

Perform 5′ single‐cell RNA sequencing (scRNA‐seq) with paired V(D)J to recover TCR sequences, quantify clonal expansion across blood and lesion and assess state transitions of dominant clonotypes. Use established algorithms such as GLIPH2 to infer shared antigen specificity groups [87]. A key prediction is that expanded lesion‐resident clones cycle through activation states without entering a stable TOX^high programme under in vivo conditions that are intermittent in antigen exposure.

### 7.5 Functional Metabolic Profiling

Quantify bioenergetics in sorted PD‐1^high CD8^+^ cells using extracellular flux assays to derive oxygen consumption and extracellular acidification parameters, ATP production rates from oxidative phosphorylation and glycolysis, and spare respiratory capacity [88]. Complement with SCENITH flow‐based profiling to map pathway dependencies at single‐cell resolution in fresh samples [89]. Report whether exhaustion‐resistant cells show preserved oxidative capacity and translation under pathway blockade.

### 7.6 Ex Vivo Perturbations That Test Each Axis

To interrogate the intermittent TCR axis, compare pulsatile anti‐CD3 stimulation (short pulses separated by rest intervals) with continuous stimulation and quantify cytotoxic readouts, induction of TOX and NR4A modules, and state shifts of dominant clonotypes. For the metabolic axis, titrate inhibitors of mTOR or mitochondrial respiration and attempt rescue with agents that enhance mitochondrial biogenesis; read out effector function together with oxygen‐consumption rate (OCR), extracellular‐acidification rate (ECAR), ATP production and spare respiratory capacity. For the IL‐15/CD28 axis, block IL‐15Rα or Janus kinase (JAK)‐STAT signalling or inhibit CD28–B7 co‐stimulation and determine whether effector competence declines with a concomitant rise in TOX‐programme scores [90]. These perturbations nominate pharmacodynamic readouts (e.g., pSTAT5 in CD8^+^ T cells, OCR/ECAR and TOX‐module scores) that can be translated into early‐phase trials.

### 7.7 Falsification Criteria and Biomarkers

The model would be falsified if PD‐1^high cytotoxic CD8^+^ T cells in lesions uniformly display TOX^high programmes with low mitochondrial fitness when measured by single‐cell multi‐omics and functional assays or if targeted blockade of IL‐15/JAK or CD28–B7 signals fails to reduce effector competence ex vivo. Positive validation would nominate biomarkers such as pSTAT5 levels, IL‐15 spatial density near PD‐1^high clones, high spare respiratory capacity and accessibility at enhancer elements near TCF7 and cytotoxic loci for future patient stratification.

## 8 Applicability to AS and Clinical Implications

Ankylosing spondylitis presents a distinctive immunologic milieu that aligns with the proposed three‐axis model. HLA‐B27 biology and the enthesis organ architecture create niches for local immune activation; human enthesis contains resident lymphocytes that respond to interleukin‐23 and γδ T cells capable of interleukin‐17 production, supporting site‐restricted stimulation [14, 15, 16]. A gut–joint axis is also evident: subclinical intestinal inflammation is frequent and can be tracked by faecal calprotectin, and an AS‐associated ileal microbial signature has been described [17, 18, 19]. Repetitive biomechanical stress at entheses provides episodic danger cues and antigen release, favouring intermittent and tissue‐confined TCR engagement rather than uniform, chronic exposure [20].

Understanding how CD8^+^ T cells resist exhaustion suggests testable therapeutic strategies in AS. Janus kinase inhibitors intercept signalling from common γ‐chain cytokines, including interleukin‐15, which can provide survival and metabolic support to cytotoxic T cells; consistent with this mechanism, JAK inhibition has shown clinical benefit in axial spondyloarthritis [90]. Candidate pharmacodynamic markers derived from our roadmap include pSTAT5 in lesion‐derived CD8^+^ T cells, IL‐15 spatial density near PD‐1^high aggregates and spare respiratory capacity. In phase 3 studies, tofacitinib improved signs and symptoms of active ankylosing spondylitis versus placebo, and upadacitinib achieved rapid responses with sustained efficacy across radiographic and non‐radiographic cohorts [91, 92, 93]. These data support targeting cytokine networks that sustain exhaustion‐resistant CD8^+^ T cell pools and motivate hypothesis‐driven trials of agents that modulate the interleukin‐15–JAK–STAT5 axis or CD28–B7 co‐stimulation in carefully selected patients. Safety considerations reported with JAK inhibition in other immune diseases should be prospectively monitored in AS [94].

Illustrative examples for JAK inhibition include upadacitinib in radiographic and non‐radiographic axSpA: SELECT‐AXIS 1 (phase 2/3; long‐term extension to 2 years; NCT03178487) [95] and SELECT‐AXIS 2 (phase 3; NCT04169373) [92], as well as tofacitinib (phase 3; NCT03502616) [91]. In addition, filgotinib has progressed to a phase 3 axSpA programme (OLINGUITO; e.g., NCT05785611; EU CTIS 2022–501 354–10‐01) with positive topline results reported in July 2025. For IL‐15 antagonism, active development is underway in related immune conditions, including ordesekimab/PRV‐015 in non‐responsive coeliac disease (NCT04424927; prior AMG‐714 study NCT02637141) and CALY‐002 in coeliac disease/eosinophilic oesophagitis (NCT04593251) [96, 97]. To our knowledge, no interventional trials of IL‐15 antagonists in axial spondyloarthritis were registered at the time of writing.

## 9 Future Directions

### 9.1 Validating the Core Molecular Mechanisms

First, it is crucial to validate the core molecular mechanisms proposed in our model. This will involve using advanced techniques like spatial transcriptomics to map the niches where these cells reside and identify their sustaining signals [85]. Furthermore, detailed epigenetic analyses, such as assays for transposase‐accessible chromatin using sequencing (ATAC‐seq) on sorted PD‐1^+^TOX^−^ cells, are needed to define their unique chromatin landscape and reveal novel regulatory targets. Finally, the causative roles of the IL‐15/CD28 axis and metabolic pathways must be confirmed using AS‐relevant preclinical models through genetic or pharmacological interventions.

### 9.2 Translational Research and Biomarker Development

A key goal is to determine whether the frequency or functional signature of circulating or synovial exhaustion‐resistant CD8^+^ T cells can serve as biomarkers for disease activity, structural progression or treatment response. Prospective, biomarker‐enriched studies should link target engagement to clinical outcomes. Candidate readouts include pSTAT5 in lesion‐derived CD8^+^ T cells, spatial density of interleukin‐15–producing niches near PD‐1^high CD8^+^ aggregates, spare respiratory capacity and ATP production rates from extracellular flux analysis, and transcriptomic module scores such as TOX‐ and TCF7‐associated programmes with paired TCR tracking. Monitoring how this compartment changes under approved and experimental therapies will clarify mechanisms of action and resistance.

### 9.3 Developing Novel Therapeutic Strategies

This framework also inspires the development of novel therapeutic strategies. Beyond just blocking inflammatory outputs, future approaches could aim to reprogram the pathogenic T cells themselves. For instance, exploring ways to target the supporting axes, such as with IL‐15 receptor antagonists, represents a direct application of our model. Alternatively, strategies aimed at therapeutically inducing exhaustion, perhaps by modulating the expression or activity of key transcription factors like TOX [28], could offer a way to selectively silence these pathogenic cells without broad immunosuppression.

## 10 Outlook

The conceptual framework of ‘CD8^+^ T‐cell exhaustion‐resistant’ provides a novel lens through which to understand the persistent immune activation and structural progression in ankylosing spondylitis. This theory brings CD8^+^ T cells to the centre of AS immunopathology, complementing the classical Th17/IL‐17 axis. While previous models often emphasised HLA‐B27 misfolding or innate immune activation, our review repositions HLA‐B27 as a sustained antigen‐presenting molecule that expands exhaustion‐resistant CD8^+^ clones, thereby fuelling adaptive immune dysregulation. The documented flares of arthritis in AS patients undergoing checkpoint inhibitor therapy underscore the clinical relevance of this mechanism.

In therapeutic terms, the concept of exhaustion‐resistant encourages a shift from rejuvenating immunity—as in cancer—to promoting controlled attrition of autoreactive CD8^+^ T cells. Strategies such as driving TOX/NR4A expression, locally enhancing PD‐1 signalling or disrupting IL‐15–mediated metabolic support hold promise for selectively silencing these pathogenic T cells without compromising systemic defence. JAK inhibitors, already efficacious in AS, may partly exert their effects by attenuating the very cytokine circuits that sustain exhaustion‐resistant.

Still, this model remains theoretical and demands further empirical support. Future studies should dissect the epigenetic and metabolic signatures of exhaustion‐resistant CD8^+^ T cells, clarify their interaction with the osteoimmune microenvironment, and evaluate targeted interventions in animal models. Importantly, the spatial organisation and niche‐specific signals that foster this resistant state warrant close investigation.

In summary, exhaustion‐resistant CD8^+^ T cells may constitute a missing link between persistent inflammation, structural damage and immunotherapy refractoriness in AS. Anchored in both molecular insight and clinical observation, this framework may not only refine disease taxonomy but may also help guide the next generation of immune‐modulating therapies in spondyloarthritides.

## Author Contributions

**Xuhong Zhang:** conceived the structure of the review and conducted the literature search. **Lu Jia** and **Xueni Lin:** drafted the initial manuscript and prepared the figures. **Lamei Zhou:** contributed to the critical revision of the manuscript. All authors read and approved the final version of the manuscript.

## Disclosure

The authors have nothing to report.

## Ethics Statement

The authors have nothing to report.

## Conflicts of Interest

The authors declare no conflicts of interest.

## Data Availability

Data sharing not applicable to this article as no datasets were generated or analysed during the current study.

## References

[1] J. D. Taurog , A. Chhabra , and R. A. Colbert , “Ankylosing Spondylitis and Axial Spondyloarthritis,” New England Journal of Medicine 374 (2016): 2563–2574. 27355535 10.1056/NEJMra1406182

[2] M. A. Brown , T. Kenna , and B. P. Wordsworth , “Genetics of Ankylosing Spondylitis—Insights Into Pathogenesis,” Nature Reviews Rheumatology 12 (2016): 81–91. 26439405 10.1038/nrrheum.2015.133

[3] P. Atagunduz , H. Appel , W. Kuon , et al., “HLA–B27–Restricted CD8+ T Cell Response to Cartilage‐Derived Self Peptides in Ankylosing Spondylitis,” Arthritis and Rheumatism 52 (2005): 892–901, 10.1002/art.20948. 15751060

[4] R. A. Colbert , T. M. Tran , and G. Layh‐Schmitt , “HLA‐B27 Misfolding and Ankylosing Spondylitis,” Molecular Immunology 57 (2014): 44–51. 23993278 10.1016/j.molimm.2013.07.013 PMC3857088

[5] I. Wong‐Baeza , A. Ridley , J. Shaw , et al., “KIR3DL2 Binds to HLA‐B27 Dimers and Free H Chains More Strongly Than Other HLA Class I and Promotes the Expansion of T Cells in Ankylosing Spondylitis,” Journal of Immunology 190 (2013): 3216–3224. 10.4049/jimmunol.1202926 PMC3736094 23440420

[6] K. Pavelka , A. J. Kivitz , E. Dokoupilova , et al., “Secukinumab 150/300 Mg Provides Sustained Improvements in the Signs and Symptoms of Active Ankylosing Spondylitis: 3‐Year Results From the Phase 3 measure 3 Study,” ACR Open Rheumatology 2 (2020): 119–127. 31957970 10.1002/acr2.11102 PMC7011421

[7] R. Micheroli , S. Kissling , K. Bürki , et al., “Sacroiliac Joint Radiographic Progression in Axial Spondyloarthritis Is Retarded by the Therapeutic Use of TNF Inhibitors: 12‐Year Data From the SCQM Registry,” RMD Open 8 (2022): e002551. 36270744 10.1136/rmdopen-2022-002551 PMC9594572

[8] M. Tang , Z. Qaiyum , M. Lim , and R. D. Inman , “Single Cell Immune Profiling in Ankylosing Spondylitis Reveals Resistance of CD8+ T Cells to Immune Exhaustion,” Iscience 28 (2025): 112715. 40687839 10.1016/j.isci.2025.112715 PMC12272782

[9] V. Martini , Y. Silvestri , A. Ciurea , et al., “Patients With Ankylosing Spondylitis Present a Distinct CD8 T Cell Subset With Osteogenic and Cytotoxic Potential,” RMD Open 10 (2024): e003926. 38395454 10.1136/rmdopen-2023-003926 PMC10895246

[10] A. Baessler and D. A. A. Vignali , “T cell exhaustion,” Annual Review of Immunology 42 (2024): 179–206. 10.1146/annurev-immunol-090222-110914 38166256

[11] E. A. Komech , A. D. Koltakova , A. A. Barinova , et al., “TCR Repertoire Profiling Revealed Antigen‐Driven CD8+ T Cell Clonal Groups Shared in Synovial Fluid of Patients With Spondyloarthritis,” Frontiers in Immunology 13 (2022): 973243. 36325356 10.3389/fimmu.2022.973243 PMC9618624

[12] M. Zheng , X. Zhang , Y. Zhou , et al., “TCR Repertoire and CDR3 Motif Analyses Depict the Role of αβ T Cells in Ankylosing Spondylitis,” eBioMedicine 47 (2019): 414–426. 31477563 10.1016/j.ebiom.2019.07.032 PMC6796593

[13] M. T. Fiorillo , M. Maragno , R. Butler , M. L. Dupuis , and R. Sorrentino , “CD8^+^ T‐Cell Autoreactivity to an HLA‐B27–Restricted Self‐Epitope Correlates With Ankylosing Spondylitis,” Journal of Clinical Investigation 106 (2000): 47–53. 10880047 10.1172/JCI9295 PMC314361

[14] R. J. Cuthbert , A. Watad , E. M. Fragkakis , et al., “Evidence That Tissue Resident Human Enthesis γδT‐Cells Can Produce IL‐17A Independently of IL‐23R Transcript Expression,” Annals of the Rheumatic Diseases 78 (2019): 1559–1565. 31530557 10.1136/annrheumdis-2019-215210 PMC6837256

[15] A. Watad , H. Rowe , T. Russell , et al., “Normal Human Enthesis Harbours Conventional CD4+ and CD8+ T Cells With Regulatory Features and Inducible IL‐17A and TNF Expression,” Annals of the Rheumatic Diseases 79 (2020): 1044–1054. 32404344 10.1136/annrheumdis-2020-217309 PMC7392498

[16] J. P. Sherlock , B. Joyce‐Shaikh , S. P. Turner , et al., “IL‐23 Induces Spondyloarthropathy by Acting on ROR‐γt+ CD3+CD4−CD8− Entheseal Resident T Cells,” Nature Medicine 18 (2012): 1069–1076. 10.1038/nm.2817 22772566

[17] C. Mazeda , S. P. Silva , C. Pinto Oliveira , S. Azevedo , and A. Barcelos , “GIScaSpA—Study of Subclinical Gut Involvement in Axial Spondyloarthritis,” Rheumatology Advances in Practice 8, no. 1 (2024): rkae016. 38414917 10.1093/rap/rkae016 PMC10898325

[18] Y. Ma , D. Fan , S. Xu , et al., “Calprotectin in Spondyloarthritis: A Systematic Review and Meta‐Analysis,” International Immunopharmacology 88 (2020): 106948. 32892074 10.1016/j.intimp.2020.106948

[19] M.‐E. Costello , F. Ciccia , D. Willner , et al., “Brief Report: Intestinal Dysbiosis in Ankylosing Spondylitis,” Arthritis and Rheumatology 67 (2015): 686–691. 25417597 10.1002/art.38967

[20] M. Benjamin , B. Moriggl , E. Brenner , P. Emery , D. McGonagle , and S. Redman , “The ‘Enthesis Organ’ Concept: Why Enthesopathies May Not Present as Focal Insertional Disorders,” Arthritis and Rheumatism 50 (2004): 3306–3313. 15476254 10.1002/art.20566

[21] F. Sallusto , J. Geginat , and A. Lanzavecchia , “Central Memory and Effector Memory T Cell Subsets: Function, Generation, and Maintenance,” Annual Review of Immunology 22 (2004): 745–763. 10.1146/annurev.immunol.22.012703.104702 15032595

[22] D. Zehn , R. Thimme , E. Lugli , G. P. de Almeida , and A. Oxenius , “‘Stem‐Like’ Precursors Are the Fount to Sustain Persistent CD8+ T Cell Responses,” Nature Immunology 23 (2022): 836–847. 35624209 10.1038/s41590-022-01219-w

[23] E. J. Wherry and M. Kurachi , “Molecular and Cellular Insights Into T Cell Exhaustion,” Nature Reviews Immunology 15 (2015): 486–499. 10.1038/nri3862 PMC4889009 26205583

[24] C. U. Blank , W. N. Haining , W. Held , et al., “Defining ‘T Cell Exhaustion’,” Nature Reviews Immunology 19 (2019): 665–674. 10.1038/s41577-019-0221-9 PMC7286441 31570879

[25] H. Wu , M. Campillo Prados , and M. Vaeth , “Metabolic Regulation of T Cell Exhaustion,” Immune Discovery 1 (2025): 10005.

[26] E. J. Wherry , S. J. Ha , S. M. Kaech , et al., “Molecular Signature of CD8+ T Cell Exhaustion During Chronic Viral Infection,” Immunity 27 (2007): 670–684. 17950003 10.1016/j.immuni.2007.09.006

[27] K. E. Pauken and E. J. Wherry , “Overcoming T Cell Exhaustion in Infection and Cancer,” Trends in Immunology 36 (2015): 265–276. 25797516 10.1016/j.it.2015.02.008 PMC4393798

[28] O. Khan , J. R. Giles , S. McDonald , et al., “TOX Transcriptionally and Epigenetically Programs CD8+ T Cell Exhaustion,” Nature 571 (2019): 211–218. 31207603 10.1038/s41586-019-1325-x PMC6713202

[29] W. Seo , C. Jerin , and H. Nishikawa , “Transcriptional Regulatory Network for the Establishment of CD8+ T Cell Exhaustion,” Experimental & Molecular Medicine 53 (2021): 202–209. 33627794 10.1038/s12276-021-00568-0 PMC8080584

[30] L. Odagiu , J. May , S. Boulet , T. A. Baldwin , and N. Labrecque , “Role of the Orphan Nuclear Receptor NR4A Family in T‐Cell Biology,” Frontiers in Endocrinology 11 (2021): 624122. 33597928 10.3389/fendo.2020.624122 PMC7883379

[31] D. L. Barber , E. J. Wherry , D. Masopust , et al., “Restoring Function in Exhausted CD8 T Cells During Chronic Viral Infection,” Nature 439 (2006): 682–687. 16382236 10.1038/nature04444

[32] M. Singer , C. Wang , L. Cong , et al., “A Distinct Gene Module for Dysfunction Uncoupled From Activation in Tumor‐Infiltrating T Cells,” Cell 166 (2016): 1500–1511.e9. 27610572 10.1016/j.cell.2016.08.052 PMC5019125

[33] J.‐C. Beltra , S. Manne , M. S. Abdel‐Hakeem , et al., “Developmental Relationships of Four Exhausted CD8+ T Cell Subsets Reveals Underlying Transcriptional and Epigenetic Landscape Control Mechanisms,” Immunity 52 (2020): 825–841.e8. 32396847 10.1016/j.immuni.2020.04.014 PMC8360766

[34] E. F. McKinney , J. C. Lee , D. R. W. Jayne , P. A. Lyons , and K. G. C. Smith , “T‐Cell Exhaustion, Co‐Stimulation and Clinical Outcome in Autoimmunity and Infection,” Nature 523 (2015): 612–616. 26123020 10.1038/nature14468 PMC4623162

[35] S. J. Im , M. Hashimoto , M. Y. Gerner , et al., “Defining CD8+ T Cells That Provide the Proliferative Burst After PD‐1 Therapy,” Nature 537 (2016): 417–421. 27501248 10.1038/nature19330 PMC5297183

[36] A. Petrelli , G. Mijnheer , D. P. H. van Konijnenburg , et al., “PD‐1+CD8+ T Cells Are Clonally Expanding Effectors in Human Chronic Inflammation,” Journal of Clinical Investigation 128 (2018): 4669–4681. 30198907 10.1172/JCI96107 PMC6159979

[37] S. C. Sasson , C. L. Gordon , S. N. Christo , P. Klenerman , and L. K. Mackay , “Local Heroes or Villains: Tissue‐Resident Memory T Cells in Human Health and Disease,” Cellular & Molecular Immunology 17 (2020): 113–122. 31969685 10.1038/s41423-019-0359-1 PMC7000672

[38] L. K. Mackay , M. Minnich , N. A. M. Kragten , et al., “Hobit and Blimp1 Instruct a Universal Transcriptional Program of Tissue Residency in Lymphocytes,” Science 352 (2016): 459–463. 27102484 10.1126/science.aad2035

[39] M. Mandour , S. Chen , and M. G. H. van de Sande , “The Role of the IL‐23/IL‐17 Axis in Disease Initiation in Spondyloarthritis: Lessons Learned From Animal Models,” Frontiers in Immunology 12 (2021): 618581. 34267743 10.3389/fimmu.2021.618581 PMC8276000

[40] R. E. Hammer , S. D. Maika , J. A. Richardson , J.‐P. Tang , and J. D. Taurog , “Spontaneous Inflammatory Disease in Transgenic Rats Expressing HLA‐B27 and Human β2m: An Animal Model of HLA‐B27‐Associated Human Disorders,” Cell 63 (1990): 1099–1112. 2257626 10.1016/0092-8674(90)90512-d

[41] G. Guggino , A. Rizzo , D. Mauro , F. Macaluso , and F. Ciccia , “Gut‐Derived CD8+ Tissue‐Resident Memory T Cells Are Expanded in the Peripheral Blood and Synovia of SpA Patients,” Annals of the Rheumatic Diseases 80 (2021): e174. 31628095 10.1136/annrheumdis-2019-216456

[42] M. A. Paley , X. Yang , L. M. Hassman , et al., “Mucosal Signatures of Pathogenic T Cells in HLA‐B*27^+^ Anterior Uveitis and Axial Spondyloarthritis,” JCI Insight 9 (2024): e174776. 39024572 10.1172/jci.insight.174776 PMC11343591

[43] H. Seo , J. Chen , E. González‐Avalos , et al., “TOX and TOX2 Transcription Factors Cooperate With NR4A Transcription Factors to Impose CD8^+^ T Cell Exhaustion,” Proceedings of the National Academy of Sciences 116 (2019): 12410–12415. 10.1073/pnas.1905675116 PMC6589758 31152140

[44] S. Goswami , H. Zhao , X. Zhang , and P. Sharma , “Epigenetic Changes in T Cells in Response to Immune Checkpoint Blockade,” Journal of Clinical Oncology 34 (2016): 11549.

[45] B. Bengsch , A. L. Johnson , M. Kurachi , et al., “Bioenergetic Insufficiencies due to Metabolic Alterations Regulated by the Inhibitory Receptor PD‐1 Are an Early Driver of CD8(+) T Cell Exhaustion,” Immunity 45 (2016): 358–373. 27496729 10.1016/j.immuni.2016.07.008 PMC4988919

[46] X. Yang , L. I. Garner , I. V. Zvyagin , et al., “Autoimmunity‐Associated T Cell Receptors Recognize HLA‐B*27‐Bound Peptides,” Nature 612 (2022): 771–777. 36477533 10.1038/s41586-022-05501-7 PMC10511244

[47] M. D. Buck , R. T. Sowell , S. M. Kaech , and E. L. Pearce , “Metabolic Instruction of Immunity,” Cell 169 (2017): 570–586. 28475890 10.1016/j.cell.2017.04.004 PMC5648021

[48] M. Biniecka , M. Canavan , T. McGarry , et al., “Dysregulated Bioenergetics: A Key Regulator of Joint Inflammation,” Annals of the Rheumatic Diseases 75 (2016): 2192–2200. 27013493 10.1136/annrheumdis-2015-208476 PMC5136702

[49] K. Zheng , X. Zheng , and W. Yang , “The Role of Metabolic Dysfunction in T‐Cell Exhaustion During Chronic Viral Infection,” Frontiers in Immunology 13 (2022):843242. 35432304 10.3389/fimmu.2022.843242 PMC9008220

[50] N. Patsoukis , K. Bardhan , P. Chatterjee , et al., “PD‐1 Alters T‐Cell Metabolic Reprogramming by Inhibiting Glycolysis and Promoting Lipolysis and Fatty Acid Oxidation,” Nature Communications 6 (2015): 6692. 10.1038/ncomms7692 PMC4389235 25809635

[51] R. V. Parry , J. M. Chemnitz , K. A. Frauwirth , et al., “CTLA‐4 and PD‐1 Receptors Inhibit T‐Cell Activation by Distinct Mechanisms,” Molecular and Cellular Biology 25 (2005): 9543–9553. 16227604 10.1128/MCB.25.21.9543-9553.2005 PMC1265804

[52] E. Hui , J. Cheung , J. Zhu , et al., “T Cell Costimulatory Receptor CD28 Is a Primary Target for PD‐1‐Mediated Inhibition,” Science 355 (2017): 1428–1433. 28280247 10.1126/science.aaf1292 PMC6286077

[53] N. E. Scharping , A. V. Menk , R. S. Moreci , et al., “The Tumor Microenvironment Represses T Cell Mitochondrial Biogenesis to Drive Intratumoral T Cell Metabolic Insufficiency and Dysfunction,” Immunity 45 (2016): 374–388. 27496732 10.1016/j.immuni.2016.07.009 PMC5207350

[54] H. Lee , S.‐H. Park , and E.‐C. Shin , “IL‐15 in T‐Cell Responses and Immunopathogenesis,” Immune Network 24 (2024): e11. 38455459 10.4110/in.2024.24.e11 PMC10917573

[55] J. Lee , K. Lee , H. Bae , et al., “IL‐15 Promotes Self‐Renewal of Progenitor Exhausted CD8 T Cells During Persistent Antigenic Stimulation,” Frontiers in Immunology 14 (2023): 1117092. 37409128 10.3389/fimmu.2023.1117092 PMC10319055

[56] G. Galletti , G. de Simone , E. M. C. Mazza , et al., “Two Subsets of Stem‐Like CD8+ Memory T Cell Progenitors With Distinct Fate Commitments in Humans,” Nature Immunology 21 (2020): 1552–1562. 33046887 10.1038/s41590-020-0791-5 PMC7610790

[57] A. O. Kamphorst , A. Wieland , T. Nasti , et al., “Rescue of Exhausted CD8 T Cells by PD‐1–Targeted Therapies Is CD28‐Dependent,” Science 355 (2017): 1423–1427. 28280249 10.1126/science.aaf0683 PMC5595217

[58] M. Schirmer , C. Goldberger , R. Würzner , et al., “Circulating Cytotoxic CD8+ CD28‐ T Cells in Ankylosing Spondylitis,” Arthritis Research & Therapy 4 (2001): 71–76. 10.1186/ar386 PMC64855 11879540

[59] N. Weng , A. N. Akbar , and J. Goronzy , “CD28− T Cells: Their Role in the Age‐Associated Decline of Immune Function,” Trends in Immunology 30 (2009): 306–312. 19540809 10.1016/j.it.2009.03.013 PMC2801888

[60] W. Du , L. Pang , Y. Ba , et al., “The Expression and Significance of CD28, Ctla‐4, CD80 and CD86 in Ankylosing Spondylitis Were Also Stimulated,” Biomedical Research 29 (2018): 580–584.

[61] C. Schütz and X. Baraliakos , “What Do We Know About Co‐Stimulatory and Co‐Inhibitory Immune Checkpoint Signals in Ankylosing Spondylitis?,” Clinical and Experimental Immunology 213 (2023): 288–300. 36883249 10.1093/cei/uxad032 PMC10570999

[62] O. Traitanon , A. Gorbachev , J. J. Bechtel , et al., “IL‐15 Induces Alloreactive CD28− Memory CD8 T Cell Proliferation and CTLA4‐Ig Resistant Memory CD8 T Cell Activation,” American Journal of Transplantation 14 (2014): 1277–1289. 24842641 10.1111/ajt.12719 PMC6083870

[63] D. Wendling , “The Gut in Spondyloarthritis,” Joint, Bone, Spine 83 (2016): 401–405. 27149918 10.1016/j.jbspin.2016.02.017

[64] F. Li , H. Liu , D. Zhang , Y. Ma , and B. Zhu , “Metabolic Plasticity and Regulation of T Cell Exhaustion,” Immunology 167 (2022): 482–494. 36088582 10.1111/imm.13575

[65] C.‐H. Chang , J. D. Curtis , L. B. Maggi, Jr. , et al., “Posttranscriptional Control of T Cell Effector Function by Aerobic Glycolysis,” Cell 153 (2013): 1239–1251. 23746840 10.1016/j.cell.2013.05.016 PMC3804311

[66] D. Liu , B. Liu , C. Lin , and J. Gu , “Imbalance of Peripheral Lymphocyte Subsets in Patients With Ankylosing Spondylitis: A Meta‐Analysis,” Frontiers in Immunology 12 (2021): 696973. 34295337 10.3389/fimmu.2021.696973 PMC8291033

[67] J. A. Smith and R. A. Colbert , “Review: The Interleukin‐23/Interleukin‐17 Axis in Spondyloarthritis Pathogenesis: Th17 and Beyond,” Arthritis and Rheumatology 66 (2014): 231–241, 10.1002/art.38291. 24504793 PMC4058712

[68] D. Mauro , F. Macaluso , S. Fasano , R. Alessandro , and F. Ciccia , “ILC3 in Axial Spondyloarthritis: The Gut Angle,” Current Rheumatology Reports 21 (2019): 37. 31197599 10.1007/s11926-019-0834-9

[69] E. Klingberg , M. K. Magnusson , H. Strid , et al., “A Distinct Gut Microbiota Composition in Patients With Ankylosing Spondylitis Is Associated With Increased Levels of Fecal Calprotectin,” Arthritis Research & Therapy 21 (2019): 248. 31771630 10.1186/s13075-019-2018-4 PMC6880506

[70] E. Lubberts , M. I. Koenders , B. Oppers‐Walgreen , et al., “Treatment With a Neutralizing Anti‐Murine Interleukin‐17 Antibody After the Onset of Collagen‐Induced Arthritis Reduces Joint Inflammation, Cartilage Destruction, and Bone Erosion,” Arthritis and Rheumatism 50 (2004): 650–659. 14872510 10.1002/art.20001

[71] F. Ciccia , G. Guggino , M. Zeng , et al., “Proinflammatory CX3CR1+CD59+Tumor Necrosis Factor‐Like Molecule 1A+Interleukin‐23+ Monocytes Are Expanded in Patients With Ankylosing Spondylitis and Modulate Innate Lymphoid Cell 3 Immune Functions,” Arthritis and Rheumatology 70 (2018): 2003–2013. 29869839 10.1002/art.40582

[72] Y. Xiong , M. Cai , Y. Xu , et al., “Joint Together: The Etiology and Pathogenesis of Ankylosing Spondylitis,” Frontiers in Immunology 13 (2022): 996103. 36325352 10.3389/fimmu.2022.996103 PMC9619093

[73] C.‐H. Koh , B.‐S. Kim , C.‐Y. Kang , Y. Chung , and H. Seo , “IL‐17 and IL‐21: Their Immunobiology and Therapeutic Potentials,” Immune Network 24 (2024): e2. 38455465 10.4110/in.2024.24.e2 PMC10917578

[74] N. Liu , H. Qin , Y. Cai , et al., “Dynamic Trafficking Patterns of IL‐17‐Producing γδ T Cells Are Linked to the Recurrence of Skin Inflammation in Psoriasis‐Like Dermatitis,” eBioMedicine 82 (2022): 104136. 35785620 10.1016/j.ebiom.2022.104136 PMC9256835

[75] Y. Qiao , Y. H. Pan , W. Ling , et al., “The Yin and Yang of Regulatory T Cell and Therapy Progress in Autoimmune Disease,” Autoimmunity Reviews 16 (2017): 1058–1070. 28778708 10.1016/j.autrev.2017.08.001

[76] M. Wang , C. Liu , A. Bond , et al., “Dysfunction of Regulatory T Cells in Patients With Ankylosing Spondylitis Is Associated With a Loss of Tim‐3,” International Immunopharmacology 59 (2018): 53–60. 29625390 10.1016/j.intimp.2018.03.032

[77] C. P. M. Verkleij , C. A. O'Neill , M. E. C. Broekmans , et al., “T‐Cell Characteristics Impact Response and Resistance to T‐Cell–Redirecting Bispecific Antibodies in Multiple Myeloma,” Clinical Cancer Research 30 (2024): 3006–3022. 38687588 10.1158/1078-0432.CCR-23-3333

[78] L. M. Francisco , P. T. Sage , and A. H. Sharpe , “The PD‐1 Pathway in Tolerance and Autoimmunity,” Immunological Reviews 236 (2010): 219–242. 20636820 10.1111/j.1600-065X.2010.00923.x PMC2919275

[79] C. S. N. Klose and D. Artis , “Innate Lymphoid Cells as Regulators of Immunity, Inflammation and Tissue Homeostasis,” Nature Immunology 17 (2016): 765–774. 27328006 10.1038/ni.3489

[80] H. C. Rath , H. H. Herfarth , J. S. Ikeda , et al., “Normal Luminal Bacteria, Especially Bacteroides Species, Mediate Chronic Colitis, Gastritis, and Arthritis in HLA‐B27/Human beta2 Microglobulin Transgenic Rats,” Journal of Clinical Investigation 98 (1996): 945–953. 8770866 10.1172/JCI118878 PMC507509

[81] M. Stoeckius , C. Hafemeister , W. Stephenson , et al., “Simultaneous Epitope and Transcriptome Measurement in Single Cells,” Nature Methods 14 (2017): 865–868. 28759029 10.1038/nmeth.4380 PMC5669064

[82] S. Chen , B. B. Lake , and K. Zhang , “High‐Throughput Sequencing of the Transcriptome and Chromatin Accessibility in the Same Cell,” Nature Biotechnology 37 (2019): 1452–1457. 10.1038/s41587-019-0290-0 PMC6893138 31611697

[83] S. Ma , B. Zhang , L. M. LaFave , et al., “Chromatin Potential Identified by Shared Single‐Cell Profiling of RNA and Chromatin,” Cell 183 (2020): 1103–1116.e20. 33098772 10.1016/j.cell.2020.09.056 PMC7669735

[84] Y. Hao , S. Hao , E. Andersen‐Nissen , et al., “Integrated Analysis of Multimodal Single‐Cell Data,” Cell 184 (2021): 3573–3587.e29. 34062119 10.1016/j.cell.2021.04.048 PMC8238499

[85] P. L. Ståhl , F. Salmén , S. Vickovic , et al., “Visualization and Analysis of Gene Expression in Tissue Sections by Spatial Transcriptomics,” Science 353 (2016): 78–82. 27365449 10.1126/science.aaf2403

[86] L. Moses and L. Pachter , “Museum of Spatial Transcriptomics,” Nature Methods 19 (2022): 534–546. 35273392 10.1038/s41592-022-01409-2

[87] M. Musvosvi , H. Huang , C. Wang , et al., “T Cell Receptor Repertoires Associated With Control and Disease Progression Following *Mycobacterium tuberculosis* Infection,” Nature Medicine 29 (2023): 258–269. 10.1038/s41591-022-02110-9 PMC9873565 36604540

[88] I. Yoo , I. Ahn , J. Lee , and N. Lee , “Extracellular Flux Assay (Seahorse Assay): Diverse Applications in Metabolic Research Across Biological Disciplines,” Molecules and Cells 47 (2024): 100095. 39032561 10.1016/j.mocell.2024.100095 PMC11374971

[89] R. J. Argüello , A. J. Combes , R. Char , et al., “SCENITH: A Flow Cytometry‐Based Method to Functionally Profile Energy Metabolism With Single‐Cell Resolution,” Cell Metabolism 32 (2020): 1063–1075.e7. 33264598 10.1016/j.cmet.2020.11.007 PMC8407169

[90] F. Ciccia , D. McGonagle , R. Thomas , et al., “JAK Inhibition and Axial Spondyloarthritis: New Steps on the Path to Understanding Pathophysiology,” Frontiers in Immunology 16 (2025): 1488357. 40103808 10.3389/fimmu.2025.1488357 PMC11913702

[91] A. Deodhar , P. Sliwinska‐Stanczyk , H. Xu , et al., “Tofacitinib for the Treatment of Ankylosing Spondylitis: A Phase III, Randomised, Double‐Blind, Placebo‐Controlled Study,” Annals of the Rheumatic Diseases 80 (2021): 1004–1013. 33906853 10.1136/annrheumdis-2020-219601 PMC8292568

[92] A. Deodhar , F. den Van Bosch , D. Poddubnyy , et al., “Upadacitinib for the Treatment of Active Non‐Radiographic Axial Spondyloarthritis (SELECT‐AXIS 2): A Randomised, Double‐Blind, Placebo‐Controlled, Phase 3 Trial,” Lancet 400 (2022): 369–379. 35908570 10.1016/S0140-6736(22)01212-0

[93] D. van der Heijde , X. Baraliakos , J. Sieper , et al., “Efficacy and Safety of Upadacitinib for Active Ankylosing Spondylitis Refractory to Biological Therapy: A Double‐Blind, Randomised, Placebo‐Controlled Phase 3 Trial,” Annals of the Rheumatic Diseases 81 (2022): 1515–1523. 35788492 10.1136/ard-2022-222608 PMC9606523

[94] S. R. Ytterberg , D. L. Bhatt , T. R. Mikuls , et al., “Cardiovascular and Cancer Risk With Tofacitinib in Rheumatoid Arthritis,” New England Journal of Medicine 386 (2022): 316–326, 10.1056/NEJMoa2109927. 35081280

[95] D. van der Heijde , I. H. Song , A. L. Pangan , et al., “Efficacy and Safety of Upadacitinib in Patients With Active Ankylosing Spondylitis (SELECT‐AXIS 1): A Multicentre, Randomised, Double‐Blind, Placebo‐Controlled, Phase 2/3 Trial,” Lancet 394 (2019): 2108–2117. 31732180 10.1016/S0140-6736(19)32534-6

[96] M.‐L. Lähdeaho , M. Scheinin , P. Vuotikka , et al., “Safety and Efficacy of AMG 714 in Adults With Coeliac Disease Exposed to Gluten Challenge: A Phase 2a, Randomised, Double‐Blind, Placebo‐Controlled Study,” Lancet Gastroenterology and Hepatology 4 (2019): 948–959. 31494096 10.1016/S2468-1253(19)30264-X

[97] A. P. Vicari , A. M. Schoepfer , B. Meresse , et al., “Discovery and Characterization of a Novel Humanized Anti‐IL‐15 Antibody and Its Relevance for the Treatment of Refractory Celiac Disease and Eosinophilic Esophagitis,” MAbs 9 (2017): 927–944. 28581883 10.1080/19420862.2017.1332553 PMC5540113

